# Supplementary material for: Polarized X-ray scattering measures molecular orientation in polymer-grafted nanoparticles
Source: Nat Commun. 2021 Aug 12;12:4896. doi: 10.1038/s41467-021-25176-4 (PMC8361200; doi:10.1038/s41467-021-25176-4)
Supplement: Supplementary file 1 — Supplementary Information [file 41467_2021_25176_MOESM1_ESM.pdf]

Supplementary Information

Polarized X-ray scattering measures molecular orientation in polymer-grafted nanoparticles

Subhrangsu Mukherjee[1], Jason K. Streit[2,3], Eliot Gann[1], Kumar Saurabh[4], Daniel F. Sunday[1], Adarsh Krishnamurthy[4], Baskar Ganapathysubramanian[4], Lee J. Richter[1], Richard A. Vaia[2], Dean M. DeLongchamp[1*]

[1]*Material Measurement Laboratory, National Institute of Standards and Technology, Gaithersburg, Maryland, 20899, USA*

[2]*Materials and Manufacturing Directorate, Air Force Research Laboratory, Wright Patterson Air Force Base, Ohio, 45433, USA*

[3]*UES, Inc., Dayton, Ohio, 45431, USA*

[4]*Department of Mechanical Engineering, Iowa State University, Ames, Iowa, 50011, USA*

*Email: dean.delongchamp@nist.gov

Table of Contents

1. P-RSoXS data from higher Mn and lower graft density sample (AuPS53): 3
2. Isotropic scattering pattern at off-resonant X-ray energies 4
3. Rotation of scattering anisotropy with electric field of incident X-rays 5
5. Calculation of r_c 6
6. Calculation of maximum graft density 8
7. Initial simulation for radial chains 9
8. Simulation for tangential chains 10
9. Best match with 2-parameter model (d = 0) 11
10. Best match with larger energy range (280 eV to 290 eV) 12
11. Dependence of scattering anisotropy on order parameter (S_0) 14
12. Dependence of scattering anisotropy on decay parameter (d) 15
13. Dependence of scattering anisotropy on aligned shell thickness (r_{aniso}) 15
14. Characterization of AuNP size 16
15. Uncertainty Quantifications (UQ) 16
16. Effect of particle number on scattering features and anisotropy 21
17. Best match for higher Mn (53 kDa) sample 23
18. Variance-covariance matrix from the fits for AuPS27 sample 24
19. Determination of PS Graft Density 25
References 27

1. P-RSoXS data from higher Mn and lower graft density sample (AuPS53)

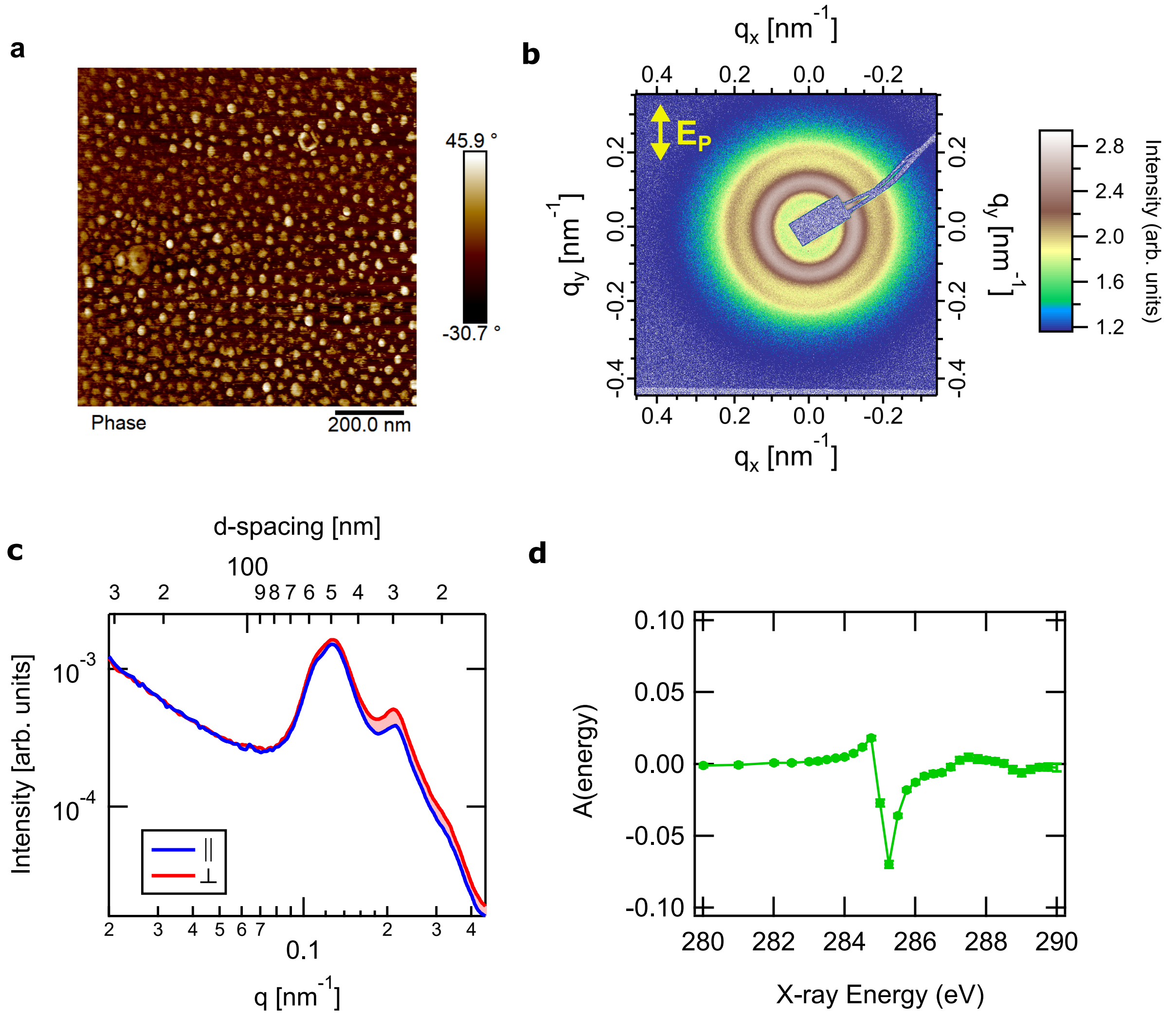

Figure S1. Microscopy of, and scattering from AuPS53 nanoparticle ultrathin film. **a**, Atomic force microscopy (AFM) phase image showing hexagonally close packed nanoparticle array. **b**, Anisotropic scattering pattern obtained using linearly polarized soft X-ray photons with the polarization vector of the electric field (**E$_P$**) along the vertical direction at a resonant energy for the aromatic rings in polystyrene (PS), 285.2 eV. **c**, Radial scattering profiles parallel (‖) and perpendicular (⊥) to incident X-ray polarization extracted from scattering pattern shown in **b**. Area of shaded region between the curves is proportional to the magnitude of scattering anisotropy. Red (blue) shading implies negative (positive) anisotropy. **d**, Anisotropy parameter (see text for

definition) calculated by integrating the scattering profiles over the experimental q-range (0.02 nm^{-1} to 0.4 nm^{-1}).

2. Isotropic scattering pattern at off-resonant X-ray energies

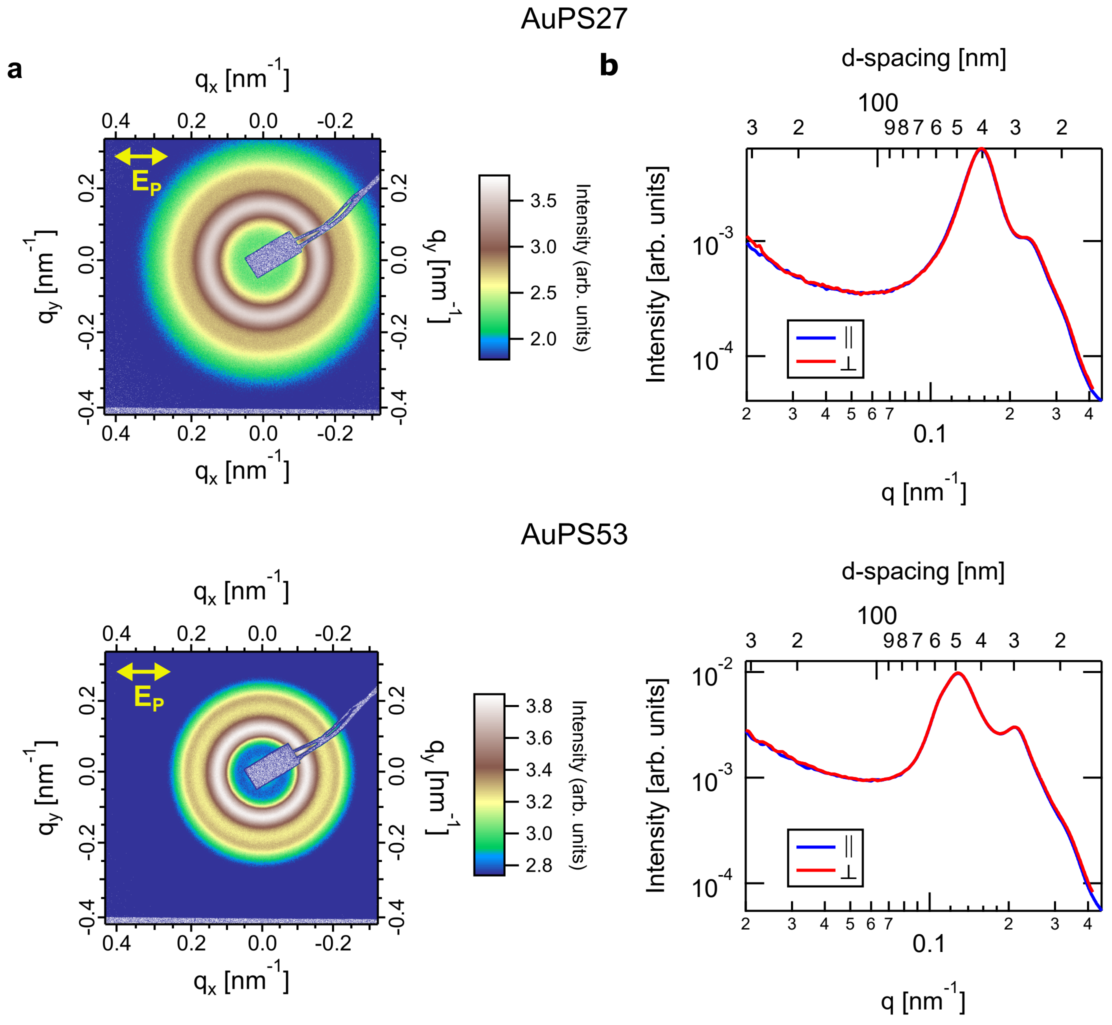

Figure S2. Isotropic scattering pattern at off-resonant X-ray energies. **a**, Scattering patterns obtained using linearly polarized soft X-ray photons with the polarization vector of the electric field (**E**$_P$) along the horizontal direction at an off-resonant energy of 270 eV for AuPS27 (Top) and AuPS53 (Bottom) samples. **b**, Radial scattering profiles parallel (∥) and perpendicular (⊥) to

incident X-ray polarization extracted from scattering pattern shown in **a** for AuPS27 (Top) and AuPS53 (Bottom) samples.

3. Rotation of scattering anisotropy with electric field of incident X-rays

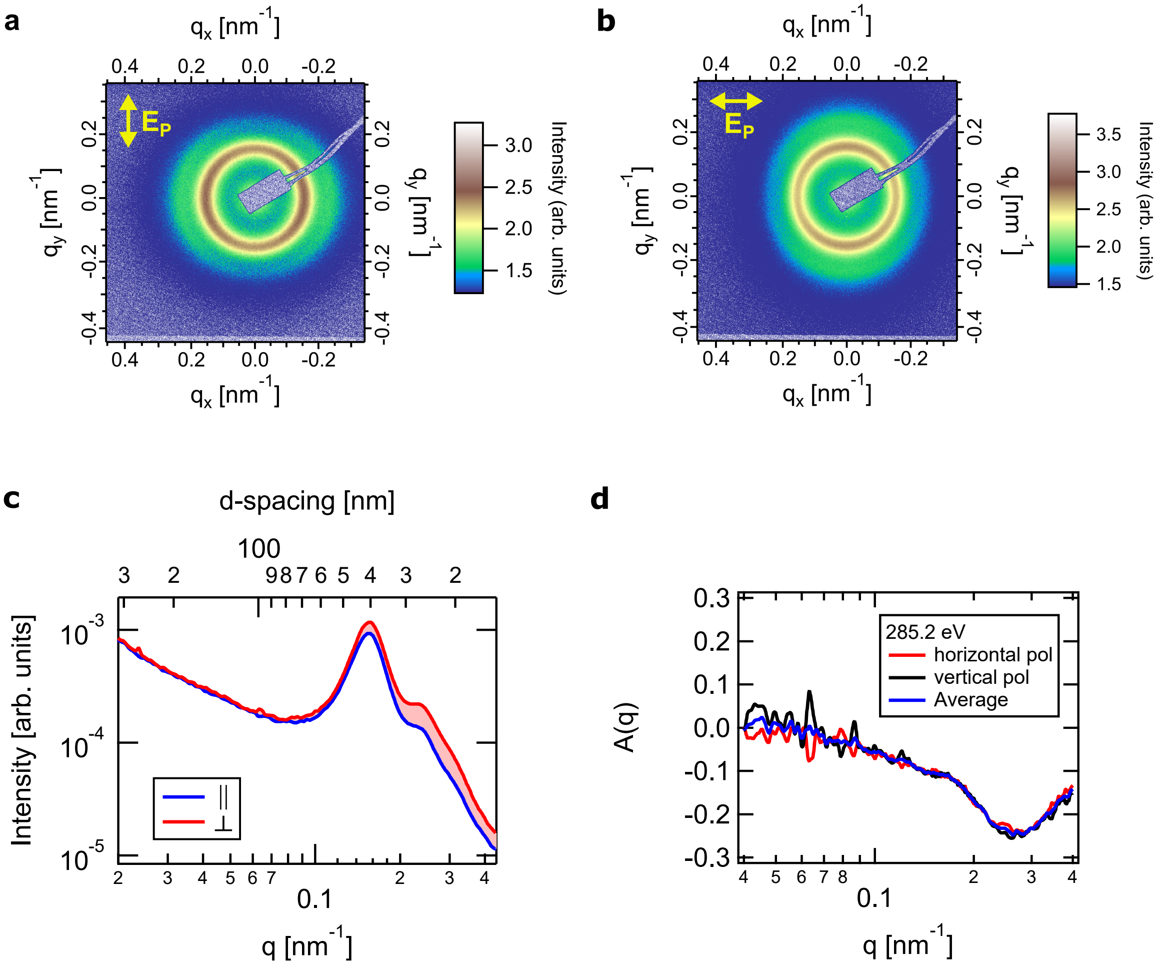

Figure S3. Rotation of scattering anisotropy with electric field of incident X-rays. Scattering pattern obtained using linearly polarized soft X-ray photons with the polarization vector of the electric field (**E**$_{\mathbf{P}}$) along the **a**, vertical, and **b**, horizontal direction at a resonant energy of 285.2 eV for AuPS27 sample. **c**, Radial scattering profiles parallel (∥) and perpendicular (⊥) to incident X-ray polarization extracted from scattering pattern shown in **b**. Area of shaded region between

the curves is proportional to the magnitude of scattering anisotropy. Red (blue) shading implies negative (positive) anisotropy. **d**, *A(q)* vs. *q* profiles from the patterns acquired with vertically and horizontally polarized X-rays, and the average result from the two polarizations.

4. Optical constants

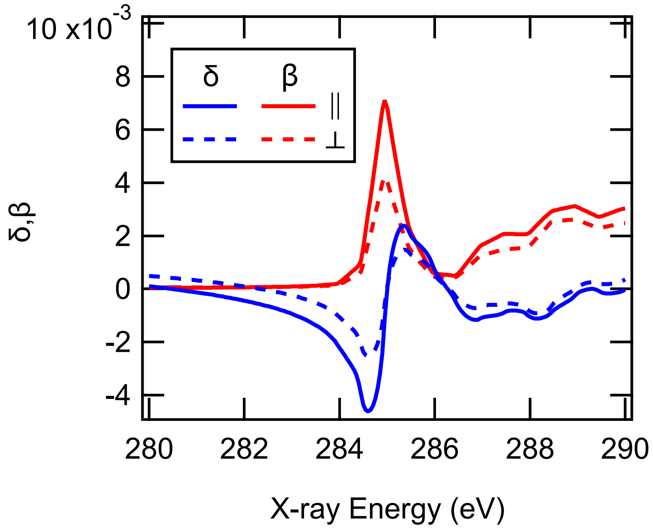

Figure S4. Anisotropic optical constants of PS. Real (δ) and imaginary part (β) of the uniaxial refractive index tensor parallel (‖) and perpendicular (⊥) to the backbone axis as illustrated in Figure 2 in main text.

5. Calculation of r_c

As given by Ohno et al.[1], the critical radius can be described by:

$$r_c = \frac{r_0\sqrt{\sigma_0^*}}{\upsilon^*}$$

where r_0 is the particle radius, σ_0^* is the dimensionless graft density, and υ^* is a rescaled excluded volume parameter, given by:

$$\sigma_0^* = \sigma a^2$$

where a is the Kuhn length and σ is the graft density in chains per area. The graft density we measure by spectroscopy is 1.2 chains/nm^2 and Kuhn length for atactic polystyrene is 1.58 nm,[2] thus

$$\sigma_0^* = 1.2\ \text{nm}^{-2}(1.58\ \text{nm})^2 = 2.99$$

The excluded volume is scaled as:

$$\upsilon^* = \frac{\upsilon}{\sqrt{4\pi}}$$

where υ is the excluded volume as defined by Daoud and Cotton[3]:

$$\upsilon = \frac{1}{2} - \chi$$

where χ is the usual Flory-Huggins interaction parameter. Because no solvent is present, we can assume $\chi = 0$ and thus $\upsilon = 0.5$.

Substituting this into the critical radius equation we get:

$$r_c = \frac{10\text{nm}\cdot\sqrt{2.99}}{(\frac{0.5}{\sqrt{4\pi}})} = \frac{10 \cdot 1.72}{(\frac{0.5}{\sqrt{4\pi}})} = \frac{10 \cdot 1.72}{0.141} = 121\ \text{nm}$$

Note other swelling excluded volume arguments, for example if we thought that swelling during casting had an effect, would deliver similar order of magnitude.

Because the contour length of our polystyrene is:

$$L = Nb = \left(\frac{27000\ \text{g/mol}}{\frac{104.1\ \text{g}}{\text{mol}\cdot\text{repeat}}}\right)\frac{0.252\ \text{nm}}{\text{repeat}} = 259\ \text{repeat} \cdot \frac{0.252\ \text{nm}}{\text{repeat}} = 65.3\ \text{nm}$$

the expectation is that the entire canopy is in the concentrated polymer brush region.

6. Calculation of maximum graft density

If we assume that crystalline syndiotactic PS represents the densest PS packing possible, then it is a reasonable model to estimate the maximum graft density. Most s-PS crystals occur as monoclinic structures where the c-parameter is the fiber axis and chain long axis.[4] There are many polymorphs, but they all have similar geometry and dimensions. The one shown below is typical; this is for the δ_e phase of s-PS, which is a crystal structure out of which the solvent molecules have been extracted, again to give us maximum packing.

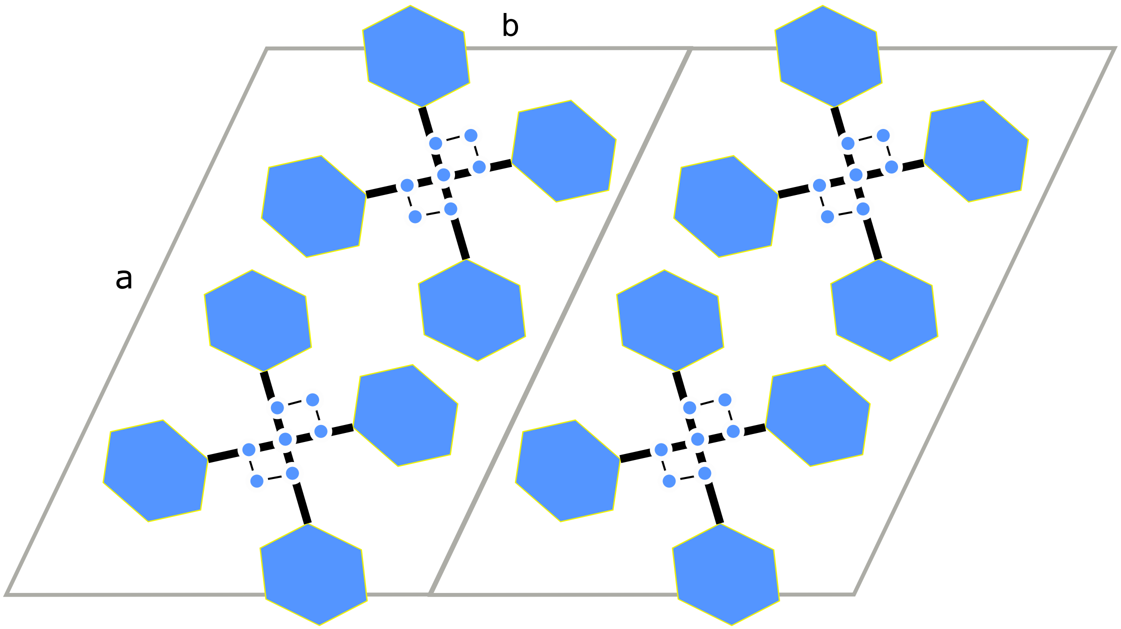

Figure S5. Packing model of the δ_e form of s-PS. Two cells along *b* are shown.

In this geometry, a = 17.4 Å, b = 11.85 Å, c (fiber axis) = 7.70 Å, and γ = 117°, with a P21/a space group. The c-axis is the long axis moving into and out of the paper normal to the plane shown. Our

concern is the chains/nm^2 within the plane shown, which only requires that we calculate the area of the a-b parallelogram, which contains 2 s-PS chains.

$$s = a \cdot b \cdot \sin(\theta) = 1.74\ \text{nm} \cdot 1.185\ \text{nm} \cdot \sin(180° - 117°) = 1.83\ \text{nm}^2$$

$$\sigma_{max} = \frac{2\ \text{chains}}{1.83\ \text{nm}^2} = 1.1\ \text{chains/nm}^2$$

7. Initial simulation for radial chains

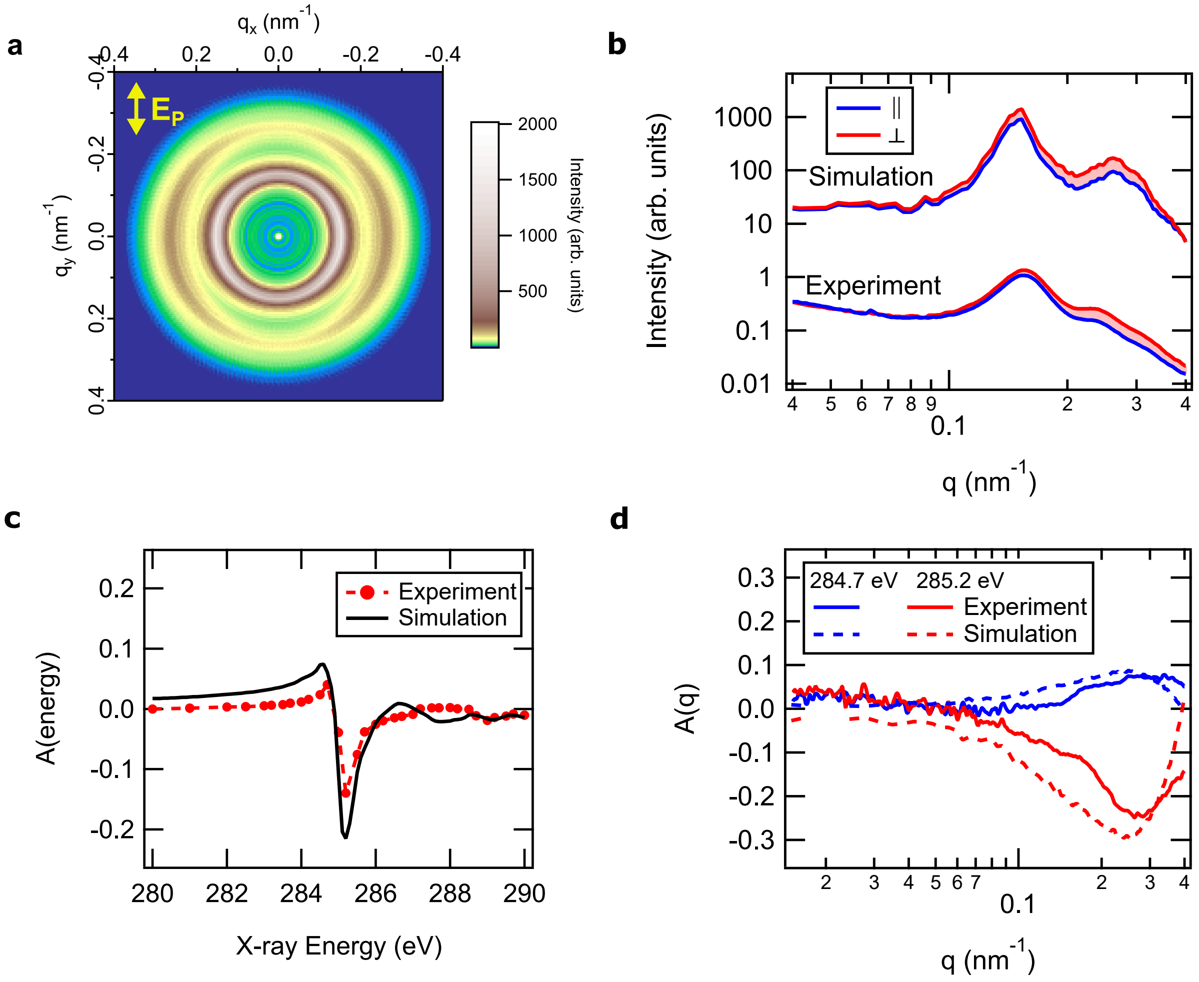

Figure S6. Initial simulation for radial chains. Simulation results for r_{aniso} = 12.5 nm, S_0 = 1, d = 2. **a**, Simulated anisotropic scattering pattern at a resonant energy of 285.2 eV with the polarization

vector of the electric field (**E**$_P$) of the incident radiation along the vertical direction. **b**, Radial scattering profiles parallel (||) and perpendicular (⊥) to incident X-ray polarization extracted from the simulated scattering pattern shown in **a**. Area of shaded region between the curves is proportional to the magnitude of scattering anisotropy. Red (blue) shading implies negative (positive) anisotropy. **c**, Experimental versus simulated anisotropy ratio (*A(energy)*) calculated by integrating the scattering profiles over the *q*-range (0.02 nm^{-1} to 0.4 nm^{-1}). **d**, Comparison of *A(q)* from experimental and simulated patterns.

8. Simulation for tangential chains

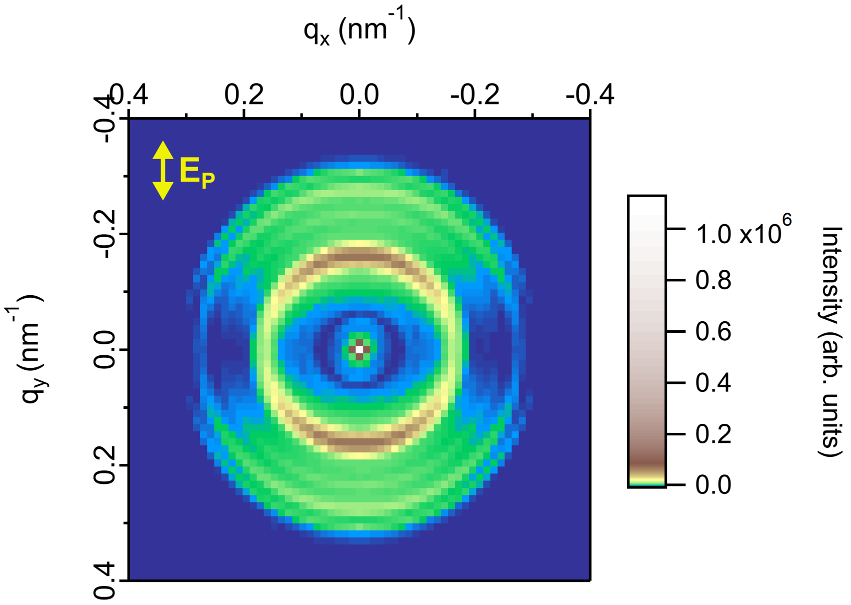

Figure S7. Simulation for tangential chains. Simulated anisotropic scattering pattern with tangential chains at a resonant energy of 285.2 eV with the polarization vector of the electric field (**E**$_P$) of the incident radiation along the vertical direction.

9. Best match with 2-parameter model ($d = 0$)

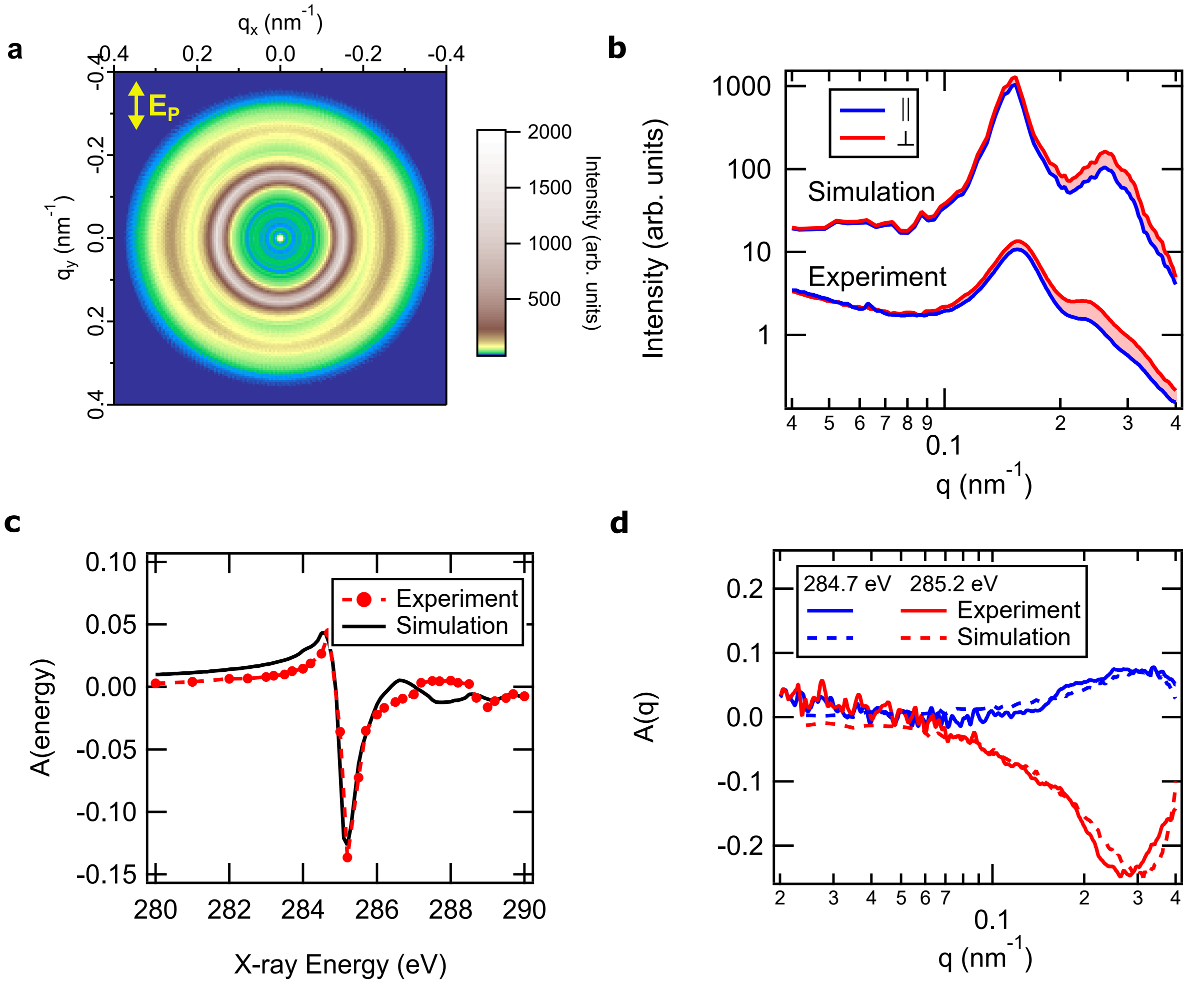

Figure S8. Best match with 2-parameter model (d = 0). Best match with a 2-parameter multi-resolution exploration using 2 energies were found for r_{aniso} = 6.8 nm, S_0 = 0.5, and decay parameter held at d = 0. **a**, Simulated anisotropic scattering pattern at a resonant energy of 285.2 eV with the polarization vector of the electric field (**E$_P$**) of the incident radiation along the vertical direction. **b**, Radial scattering profiles parallel (ǁ) and perpendicular (⊥) to incident X-ray polarization extracted from the simulated scattering pattern shown in **a**. Area of shaded region between the curves is proportional to the magnitude of scattering anisotropy. Red (blue) shading implies negative (positive) anisotropy. **c**, Experimental versus simulated anisotropy ratio

($A(energy)$) calculated by integrating the scattering profiles over the q-range (0.02 nm^{-1} to 0.4 nm^{-1}). **d**, Comparison of $A(q)$ from experimental and simulated patterns.

10. Best match with larger energy range (280 eV to 290 eV)

The values of the three parameters (r_{aniso}, S_0, and d) corresponding to the minimum of the sum of squared errors (SSE) calculated from 33 energies in the full experimental energy range (280 eV to 290 eV) are given in Table S1. The SSE minimum corresponds to the best match between $A(q)$ from experiment and simulations. The 2-D A vs q, E maps calculated from the experiment and the best match simulations are shown in Figure S9. The parameters corresponding to the case where only the two energies 284.7 eV and 285.2 eV are used for the SSE minimization are also given in Table S1 for comparison. In general, the SSE values were found to be higher and a worse match between experiment and simulations was obtained when the full experimental energy range was used compared to the two-energy case. This could be attributed to the fact that the strongest anisotropy was measured at the two resonant energies mentioned above and at all other energies the magnitude of the anisotropy is extremely low if not negligible. The experimental noise from these energies with weaker anisotropy most likely contributes to the higher SSE and causes the slight mismatch of the optimal values listed in Table S1. Nevertheless, the reasonable match between the two sets of parameters shows the robustness of our approach and results.

Table S1. Best-fit values of r_{aniso}, S_0 and d obtained from fitting the experimental and simulated $A(q)$ over the full experimental energy range (33 energies between 280 eV and 290 eV), and the case where $A(q)$ at only two resonant energies (284.7 eV and 285.2 eV) were considered. Fits were performed across 275 models having different parameters (r_{aniso}, S_0, and d) for the anisotropic corona.

Sample	r_{aniso} (nm)		S_0		d	
	33 energies	2 energies	33 energies	2 energies	33 energies	2 energies
AuPS27	7.5	7.5	0.6	0.6	0.5	1.5
AuPS53	10	10	0.3	0.4	2	2

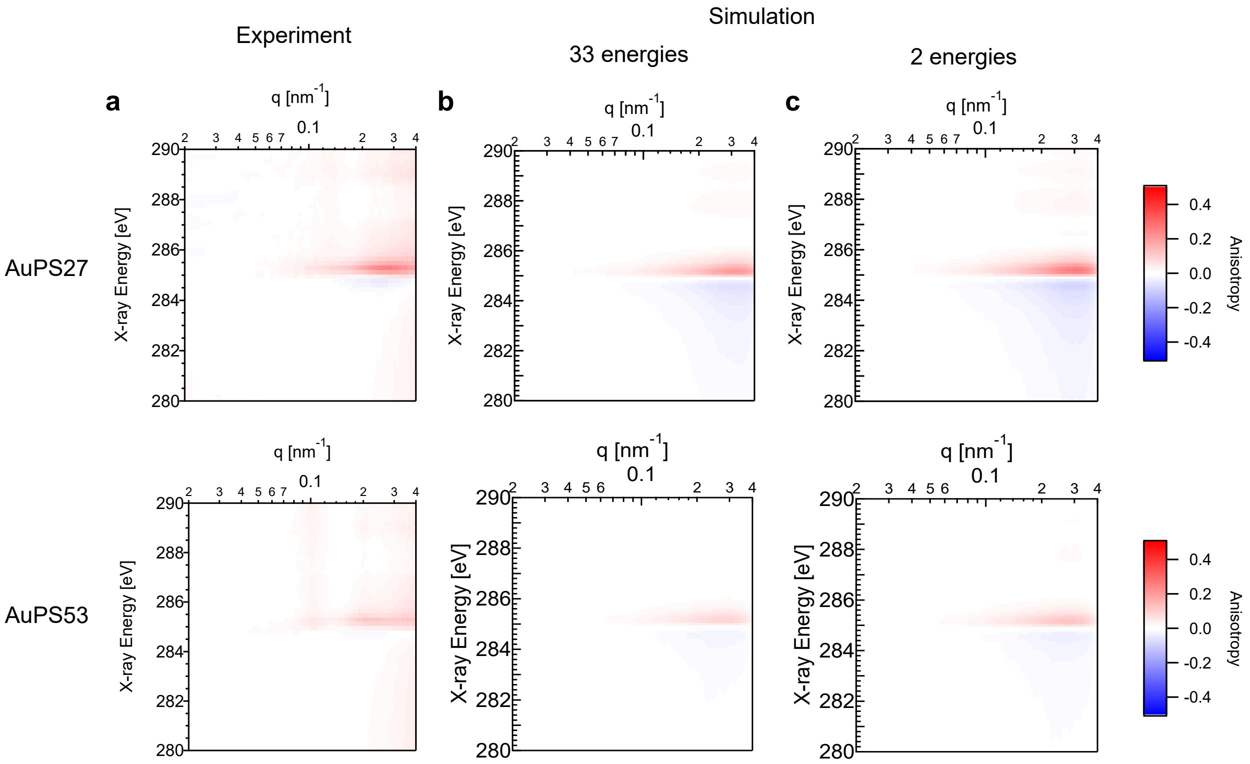

Figure S9. 2-D A vs q, E maps calculated from **a**, experimental; simulated scattering profiles calculated using morphology model corresponding to the best fit values obtained from fitting $A(q)$

across 275 different models at **b**, 33 energies between 280 eV and 290 eV, and **c**, 2 resonant energies (284.7 eV and 285.2 eV), for the two samples AuPS27 (top row) and AuPS53 (bottom row). The simulated scattering profiles were obtained using the morphology models corresponding to the parameters given in Table S1.

11. Dependence of scattering anisotropy on order parameter (S_0)

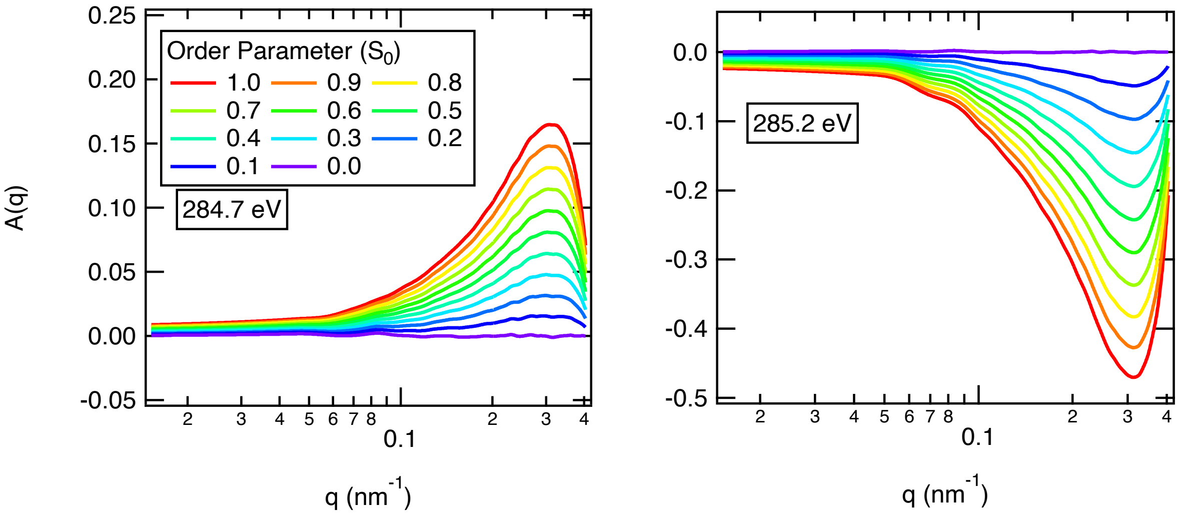

Figure S10. Dependence of scattering anisotropy on order parameter (S_0). Simulated q-dependence of anisotropy parameter ($A(q)$) for different order parameter (S_0) value with fixed r_{aniso} = 7.5 nm and d = 0.

12. Dependence of scattering anisotropy on decay parameter (*d*)

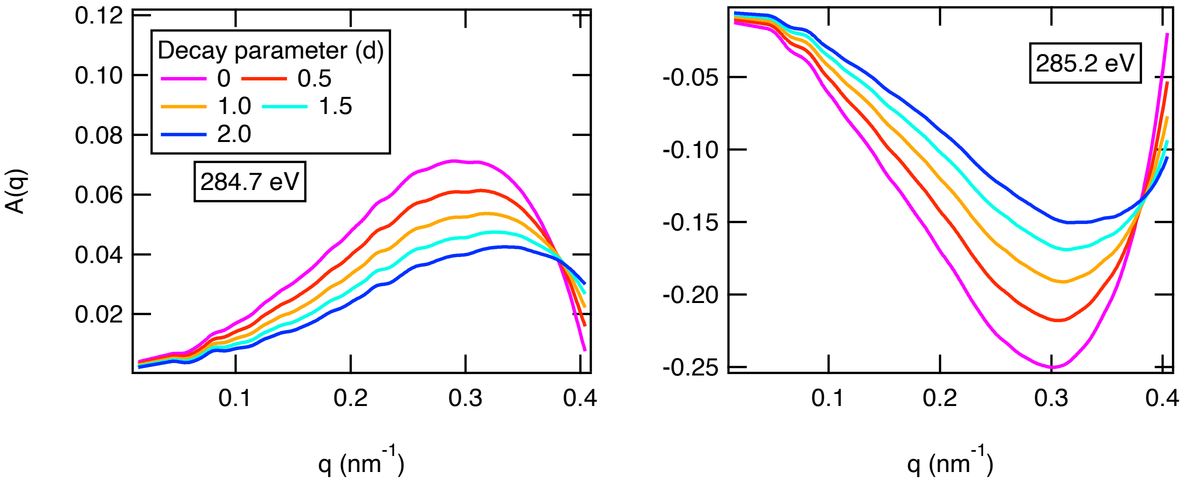

Figure S11. Dependence of scattering anisotropy on decay parameter (*d*). Simulated *q*-dependence of anisotropy parameter (*A(q)*) for different decay parameter (*d*) with fixed order parameter r_{aniso} = 7.5 nm and S_0 = 0.5.

13. Dependence of scattering anisotropy on aligned shell thickness (r_{aniso})

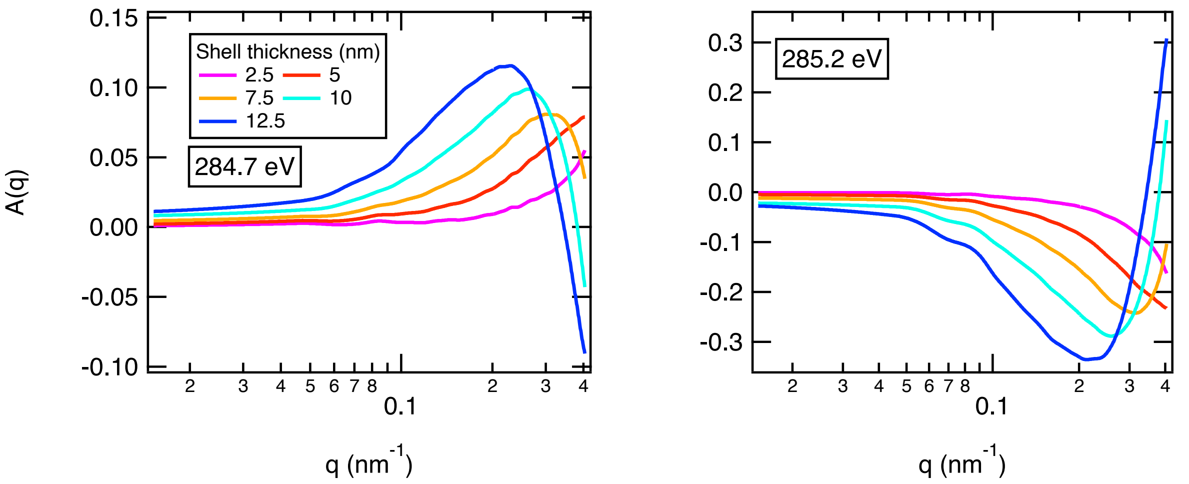

Figure S12. Dependence of scattering anisotropy on aligned shell thickness (r_{aniso}). Simulated *q*-dependence of anisotropy parameter (*A(q)*) for different r_{aniso} with fixed order parameter S_0 = 0.5 and *d* = 0.

14. Characterization of AuNP size

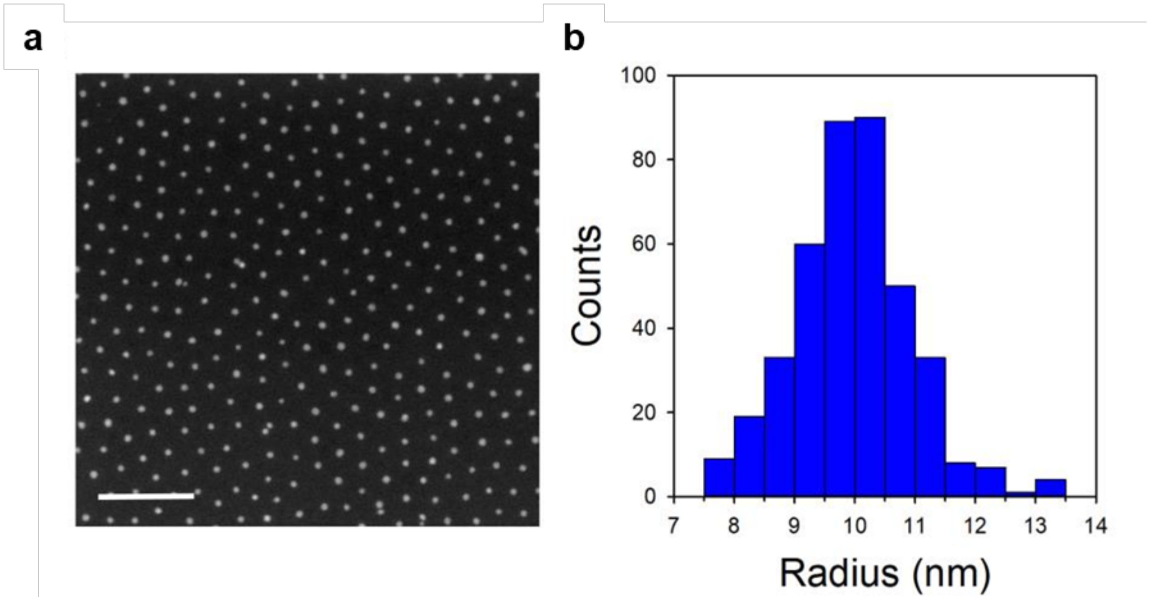

Figure S13. Characterization of AuNP size. **a**, High resolution SEM image of AuPS27 PGNs deposited on a Si substrate. Scale bar represents 250 nm. **b**, Distribution of AuNP radius obtained from image analysis of SEM images similar to **a**. The distribution gives us an average AuNP radius of (9.9 ± 1.0) nm.

15. Uncertainty Quantifications (UQ)

The understanding of the uncertainty in the response associated with the estimated parameter or model assumption is essential for understanding the material behavior. UQ enables to explore the model predictions over the range of uncertainty in parameters along with providing confidence in computational predictions. Additionally, it allows the exploration of the response of the system with the presence of noise. In this work, we deployed UQ to understand the confidence in the estimated parameters by the multi-resolution approach and understand the uncertainty in the response to the measured $A(q)$ with respect to the uncertainty in the morphology model parameters.

a. Individual variation of parameters:

First, we measured the variation in the *A(q)* in response to the variation of individual model and morphology parameters. In total, we varied the 3 model parameters (r_{aniso}, S_0, d) and 2 morphology parameters (core radius r_{np}, and surface roughness R_a). The parameter space was varied as a Gaussian random distribution with the mean set to the optimized values, and with standard deviation corresponding to ¼ of the parameter range. The mean and standard deviation for each of the parameters are shown in Table S2.

Table S2: Mean and standard deviation of parameters for UQ.

Parameter	Mean	Std. Deviation
r_{aniso}	7.4 nm	0.6 nm
S_0	0.5	0.03
d	0.4	0.1
r_{np}	10 nm	0.6 nm
R_a	1.5 nm	

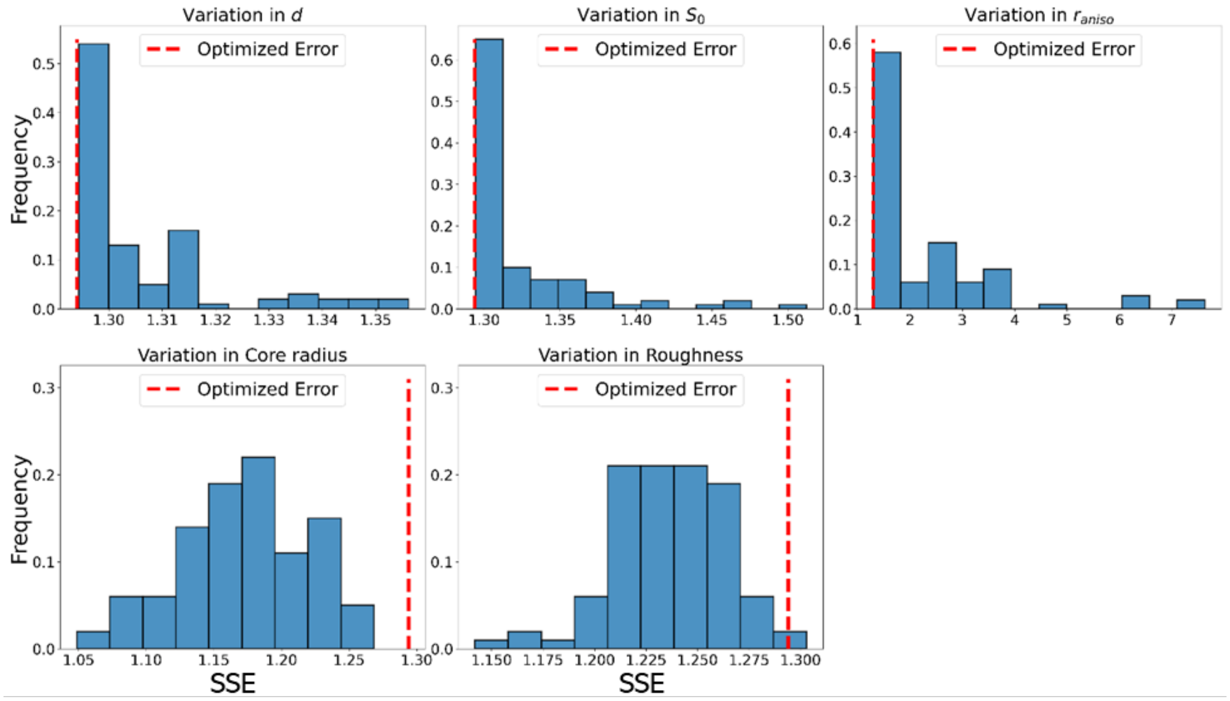

Figure S14: Normalized error frequency in *A(q)* with variation in the optimized and morphology parameters. The red line represents the optimized sum of squared errors (SSE) through the parameter sweep.

The one-sided distribution of the SSE error further enhances our confidence in the identification of the optimized parameters (r_{aniso}, S_0, d). Any variations in these parameters resulted in an increase in error.

In contrast, the variations in morphology parameters (r_{np} and R_a) resulted in a two-sided distribution of error distributions, which suggests that directly incorporating core size variation from experiments can reduce error.

a. Combined Variation of 3 parameters:

For the second UQ step, we chose to vary all the three optimized parameters (r_{aniso}, S_0, d) as a multivariate normal distribution with the same mean and standard deviation as mentioned in Table S2. Figure S15 shows the distribution of SSE. Similar to Figure S14, we observed that any deviation in the optimized parameters results in an increased error. We found that the *A(q)* vs *q* curve lies within 1 σ confidence interval.

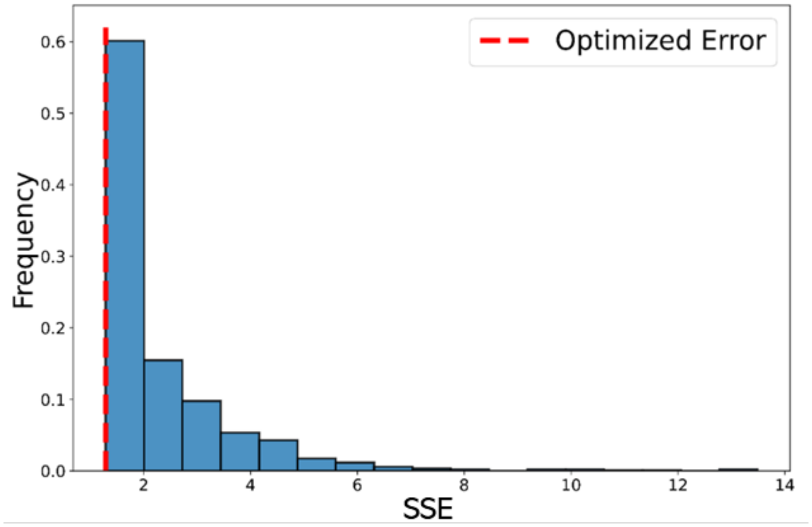

Figure S15: Normalized frequency of sum of squared errors (SSE) with combined variation of three optimized parameters (r_{aniso}, S_0, d). We observe the one-sided distribution of histogram which provides confidence in the optimized variables.

b. Combined variation of all 5 parameters

In the final step in UQ, we varied all the morphology parameters and recorded the variations in the error and *A(q)* curve. Figure S16 shows the resultant error distribution. Similar to the previous case, *A(q)* vs *q* curve (Figure S17) shows that the experimental data falls within 1 σ confidence interval.

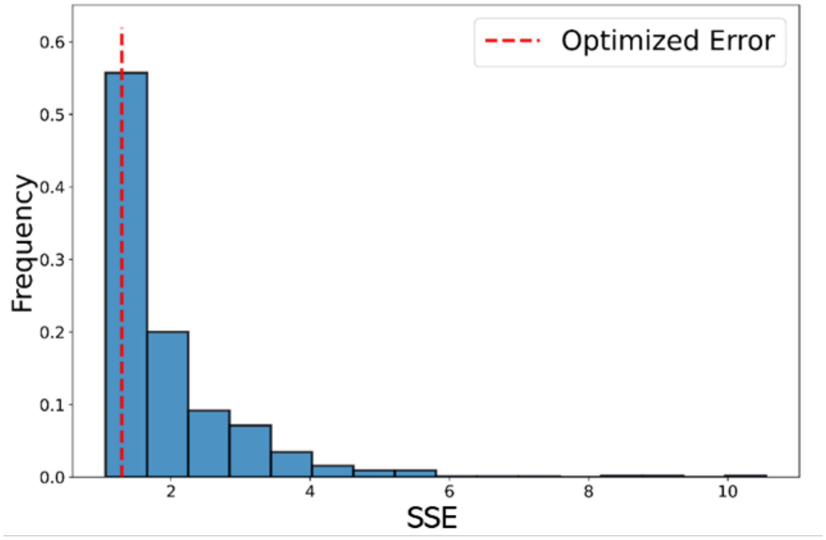

Figure S16: Normalized frequency of sum of squared errors (SSE) with variation in all 5 parameters.

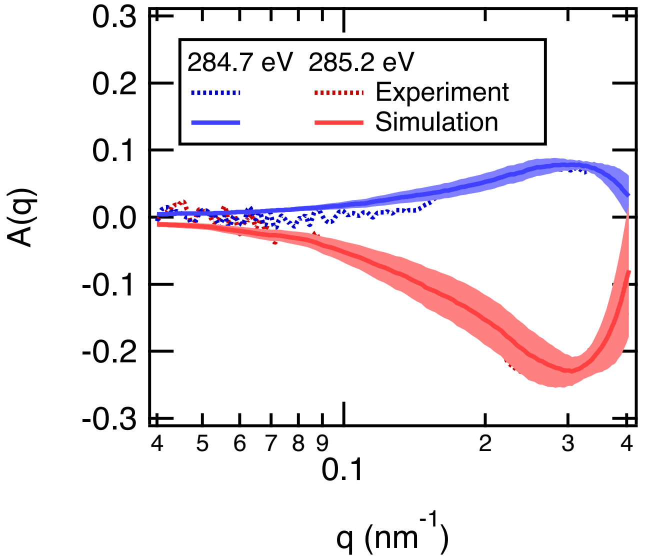

Figure S17: Plot of *A(q)* with the shaded regions showing the error from uncertainty quantification of all five parameters (r_{aniso}, S_0, d, r_{np}, R_a) in P-RSoXS simulations.

16. Effect of particle number on scattering features and anisotropy

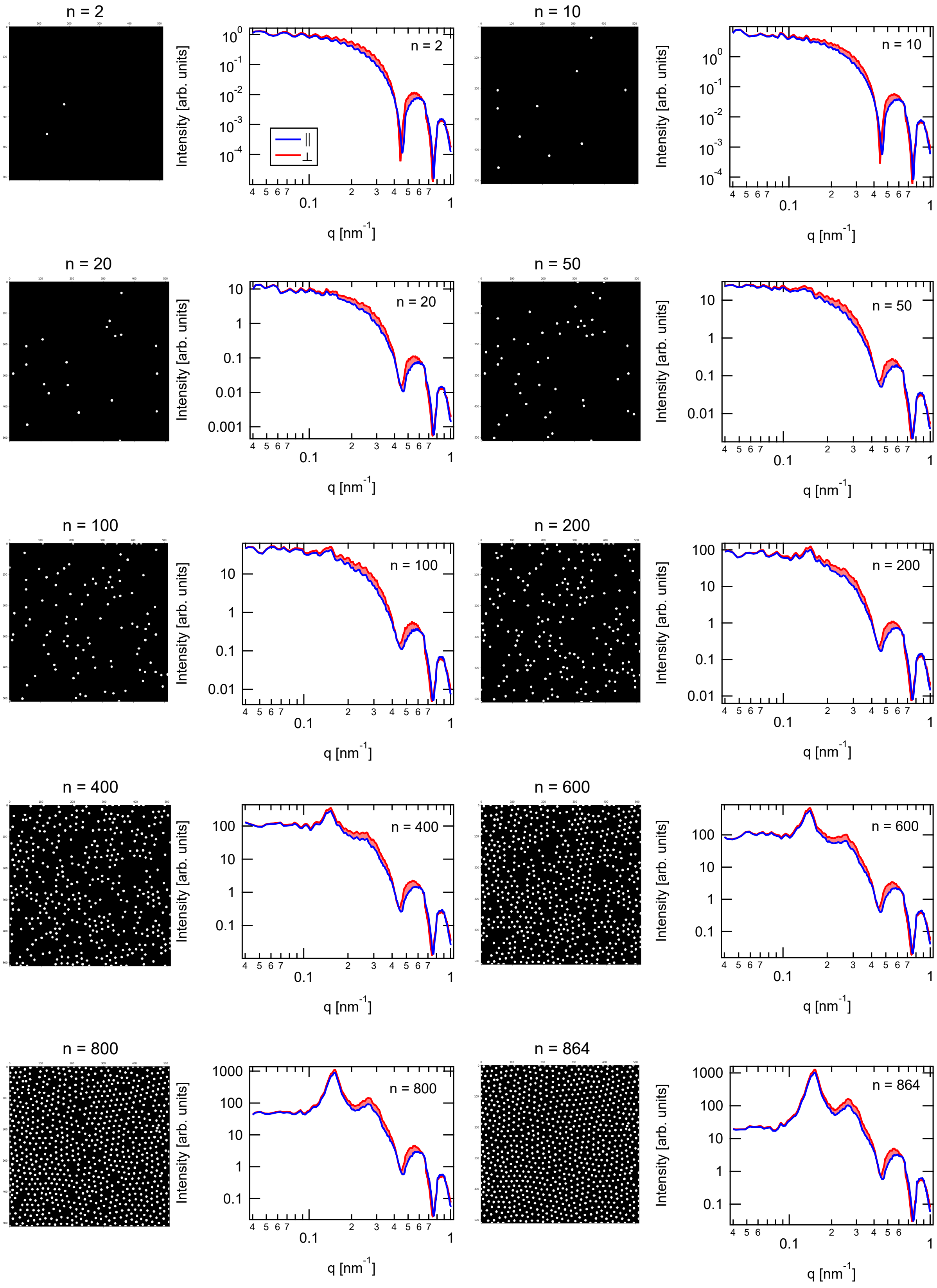

Figure S18. Simulated profiles at 285.2 eV showing the effect of gradually increasing the number of particles (n) in the morphology model on the scattering features. The particle locations (taken from Figure 1a and shown as solid white circles) correspond to the simulated morphology of the AuPS27 sample. The final morphology contains 864 particles (bottom right). The particle locations for all simulations with $n < 864$ were selected randomly from the list of coordinates obtained from Figure 1a.

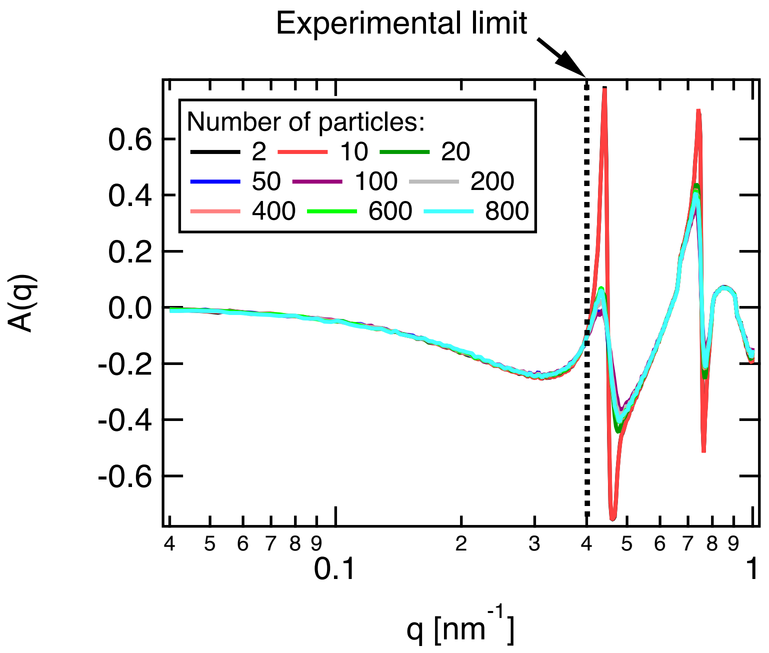

Figure S19. *A(q)* at 285.2 eV obtained from the simulated profiles for different number of particles shown in Figure S18. Experimental high-q limit is indicated with broken vertical line.

17. Best match for higher Mn (53 kDa) sample

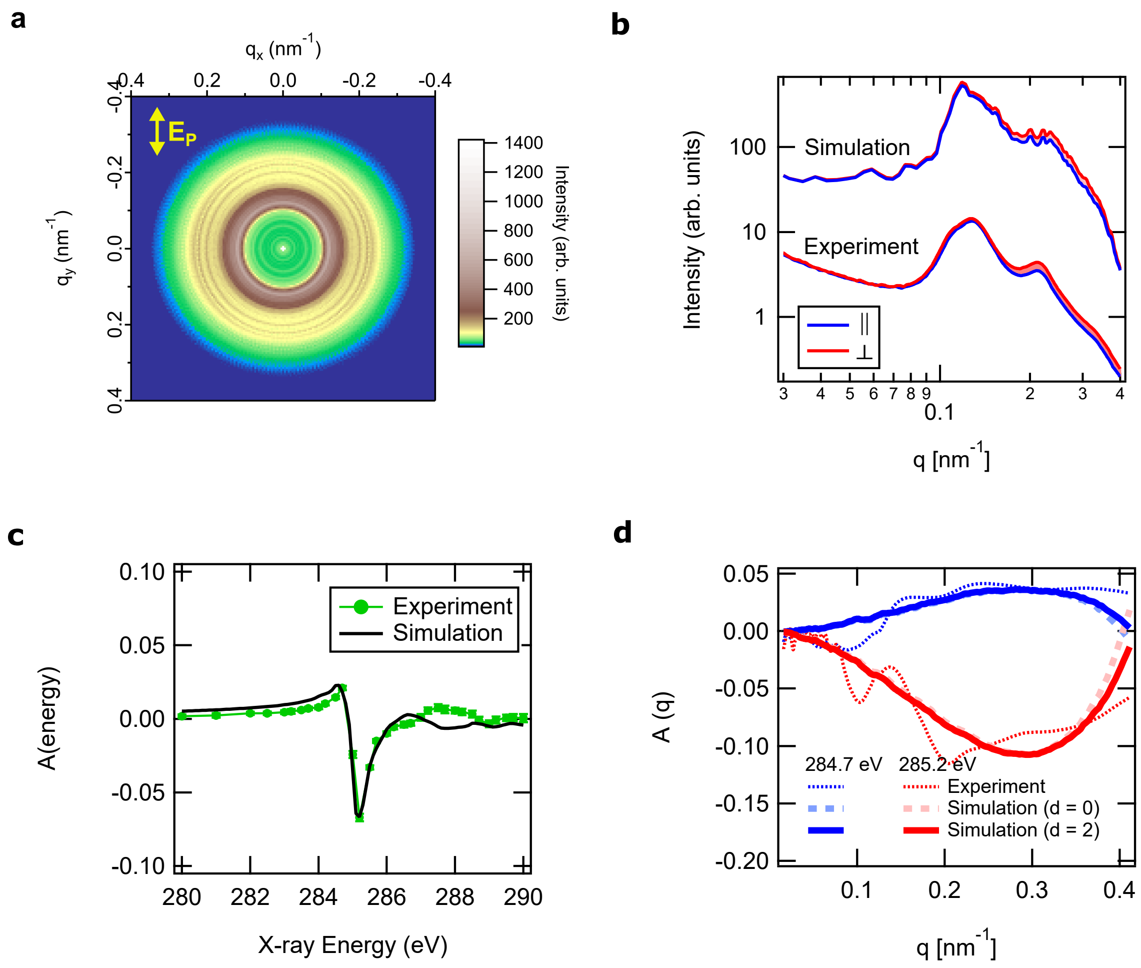

Figure S20. Best match was found for r_{aniso} = 9.3 ± 0.8 nm, S_0 = 0.4 ± 0.1, and d = 2 (fixed). **a**, Simulated anisotropic scattering pattern at a resonant energy of 285.2 eV with the polarization vector of the electric field (**E_P**) of the incident radiation along the vertical direction. **b**, Radial scattering profiles parallel (‖) and perpendicular (⊥) to incident X-ray polarization extracted from the simulated scattering pattern shown in **a**. The structure factor peak position is at lower q value suggesting a larger interparticle spacing compared to the 27 kDa sample. Area of shaded region between the curves is proportional to the magnitude of scattering anisotropy. Red (blue) shading

implies negative (positive) anisotropy. **c**, Experimental versus simulated anisotropy ratio (*A(energy)*) calculated by integrating the scattering profiles over the *q*-range (0.02 nm^{-1} to 0.4 nm^{-1}). **d**, *q*-dependence of anisotropy parameter. The higher noise in the data prevented a complete 3-parameter space exploration as was done for the AuPS27 sample. However, comparing the extremes $d = 0$ and $d = 2$ the SSE was found to be lower for the latter case. Error bars were obtained from variance-covariance matrix calculated from 90 samples corresponding to SSE threshold of 0.4.

18. Variance-covariance matrix from the fits for AuPS27 sample

The off-diagonal elements of the matrix are measure of covariances between the three parameters and the diagonal elements in the matrix are the variance values of each parameter.[5] The non-zero but unequal values of all off-diagonal elements imply that there are varying degrees of covariance between the parameters. Comparison of the magnitudes of the individual covariances between three parameters suggests a negligible covariance between S_0 and r_{aniso} compared to the considerably higher covariance between d and the other two parameters. The higher uncertainty in d compared to S_0 and r_{aniso} implies that the fits are more sensitive to variations of the latter two parameters. Overall, the above results further support our observations from the heatmaps in Figure 4.

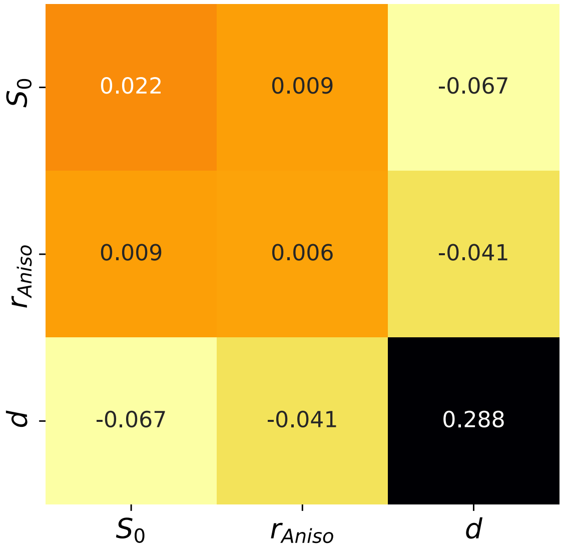

Figure S21. Variance-covariance matrix calculated from 452 samples corresponding to threshold SSE = 1.35.

19. Determination of PS Graft Density

From an average of three TGA measurements, we obtain graft densities of 1.2 ± 0.2 and 0.6 ± 0.1 for the AuPS27 and AuPS53 samples, respectively. As noted in the main text, the presence of free chains in the sample may result in an overestimation of this value. For example, if 20 % of the PS in the AuPS27 sample consists of free chains, then the true value for graft density would be 1.0 chains/nm^2.

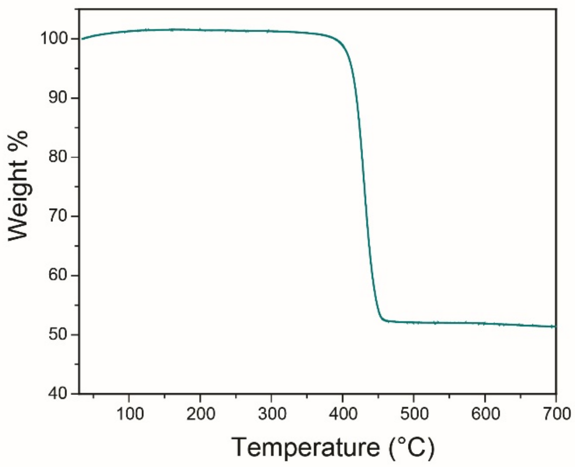

Figure S22. Experimental TGA curve for the AuPS27 sample used to determine a PS graft density of 1.2 chains/nm^2.

References

1. Ohno K, Morinaga T, Takeno S, Tsujii Y, Fukuda T. Suspensions of Silica Particles Grafted with Concentrated Polymer Brush: A New Family of Colloidal Crystals. *Macromolecules* **39**, 1245-1249 (2006).

2. Spyriouni T, Tzoumanekas C, Theodorou D, Müller-Plathe F, Milano G. Coarse-Grained and Reverse-Mapped United-Atom Simulations of Long-Chain Atactic Polystyrene Melts: Structure, Thermodynamic Properties, Chain Conformation, and Entanglements. *Macromolecules* **40**, 3876-3885 (2007).

3. Daoud M, Cotton JP. Star shaped polymers : a model for the conformation and its concentration dependence. *Journal de Physique* **43**, 531-538 (1982).

4. Gowd EB, Tashiro K, Ramesh C. Structural phase transitions of syndiotactic polystyrene. *Progress in Polymer Science* **34**, 280-315 (2009).

5. Heckert NA*, et al. Handbook 151: NIST/SEMATECH e-Handbook of Statistical Methods*. National Institute of Standards and Technology (U.S.) http://www.itl.nist.gov/div898/handbook/index2.htm (2002).
